# Identification of collagen-induced arthritis loci in aged multiparous female mice

**DOI:** 10.1186/ar1901

**Published:** 2006-02-14

**Authors:** Maria Liljander, Mary-Ann Sällström, Sara Andersson, Åsa Andersson, Rikard Holmdahl, Ragnar Mattsson

**Affiliations:** 1 Lund Transgenic Core Facility, Lund University, Lund, Sweden; 2 Department of Pharmacology, The Danish University of Pharmaceutical Sciences, Copenhagen, Denmark; 3 Medical Inflammation Research, Lund University, Lund, Sweden

## Abstract

Collagen-induced arthritis in mice is one of the most commonly used autoimmune experimental models, with many similarities to rheumatoid arthritis. Since collagen-induced arthritis is a complex polygenic disease there is a need for identification of several major disease-controlling genes. Because rheumatoid arthritis particularly affects aged women, we have in the present study identified new genetic regions critical for collagen-induced arthritis by studying aged female mice of a cross between NFR/N and B10.Q (H-2^q ^haplotype). The mice in the present study had different reproductive histories, which did not significantly affect the onset, incidence or severity of the disease. A total of 200 female mice were used in a total genome-wide screening with 125 microsatellite markers. We found one new significant quantitative trait locus affecting the arthritis incidence, severity and day of onset on chromosome 11 (denoted *Cia40*), which colocalizes with a locus controlling pregnancy failure. Furthermore, a quantitative trait locus of suggestive significance associated with the incidence, severity and day of onset was identified on chromosome 1. Finally, a suggestively significant quantitative trait locus associated with collagen type II antibody titers was identified on chromosome 13. This study indicates that several gene loci control arthritis in aged multiparous females, and that at least one of these loci coincides with pregnancy failure.

## Introduction

The similarities between rheumatoid arthritis (RA) in man and collagen-induced arthritis (CIA) in mice are well established, although differences in the disease pattern exist [1-3]. Characteristic of RA is the fact that women of reproductive age are more susceptible to the disease than men [4]. It has also been reported that 75% of female patients (23 out of 31 females) with RA exhibit clinical remission during the course of pregnancy [5]. On the other hand, it has been suggested that the onset of RA is postponed by parity. The risk of onset is reduced during pregnancy, while in the first year postpartum the chance of onset is increased [6,7]. The results of a recent study of RA in women, however, indicate that pregnancy history does not increase the incidence of the disease later in life [8].

Adequate animal models are useful tools for the identification of genes involved in complex human diseases. CIA in mice shares both immunological and pathological characteristics with human RA and is one of the most used models for the identification of genes and mechanisms involved in arthritis. The incidence of CIA is sex dependent, like the incidence in RA, although different species and different variants of the disease could lead to both male and female predominance. Male mice are more often affected than females. Gender differences in CIA susceptibility are dependent on many factors, including genetic, hormonal and behavioral influences [9]. However, isolated factors are remarkably consistent between RA and the different animal models [3,9]. Pregnancy in mice, like pregnancy in women, normally causes remission of arthritis [5,10], while exacerbation often occurs postpartum [7,10,11]. Pregnancy-induced remission of CIA in mice appears to be caused by the increase in estrogen levels, while the postpartum exacerbation can be explained by the dramatic drop in estrogen [10], possibly together with increased prolactin levels [11]. To what extent the pregnancy history (parity) affects the incidence of CIA later in life has, to our knowledge, not previously been studied. Importantly, RA occurs predominantly in women, many of which have had several children, and it is therefore appropriate to also mimic this situation using animal models.

In the present paper we have analyzed female mice that previously had undergone a reproductive study (Liljander M, Sällström MA, Andersson S, Wernhoff P, Andersson Å, Holmdahl R, Mattsson R, unpublished data). A total of 200 female mice (ten months of age) of the N2 backcross between NFR/N and C57BL/B10.Q (B10.Q) were used to analyze the genetic control of CIA in aged females with a multiparous history.

## Materials and methods

### Mice

Inbred NFR/N mice were originally obtained from the National Institute of Health (Bethesda, MD, USA) and the B10.Q mice were originally bought from The Jackson Laboratory (Bar Harbor, ME, USA). (B10Q × NFR/N)F1 hybrids and (B10.Q × NFR/N) × B10.Q N2 mice were bred in individual ventilated cages in the BMC Barrier animal facility and at the animal house of the Department of Pathology, Lund University, Sweden. The animals were fed *ad libitum *with standard rodent chow (LAB FOR R36, irradiated breeding food for rats and mice; Lactamin AB, Stockholm, Sweden) and water in a photoperiod of 12 hours:12 hours light:dark. The mice used in the present study had clean health monitoring protocols according to the Federation of European Laboratory Animal Sciences Association recommendations. Ethical permissions were M125-04 (embryo transfer) and M290-03 (reproduction and arthritis).

### Experimental design

A total of 200 female mice (approximately ten months old) of N2 backcross (B10.Q × NFR/N) × B10.Q were used. The mice had earlier undergone a reproductive study where each animal had four mating opportunities. First, the female mice were allowed to mate twice with B10.RIII males (allogeneic mating by means of MHC (major histocompatibility complex) and finally to mate twice with B10.Q males (syngeneic mating by means of MHC). After four possible pregnancies CIA was induced to the female mice.

### Induction and evaluation of CIA

To induce CIA, the mice were immunized subcutaneously at the base of the tail with 100 μg rat collagen type II (CII) emulsified in 0.1 M acetic acid combined with an equal amount of complete Freud's adjuvant (Difco Laboratories, Detroit, MI, USA). A booster injection containing 50 μg CII emulsified in 0.1 M acetic acid combined with an equal amount of Freud's incomplete adjuvant (Difco Laboratories) was given after 30 days. The clinical scoring of arthritis commenced 25 days after the first immunization. Animals were examined for clinical signs of CIA three times per week and were graded on a 12-point scale. The arthritis index was assigned to each mouse using the following criteria: 0 = no visible sign of arthritis, 1 = redness and swelling in a single joint, 2 = inflammation in multiple joints, and 3 = severe inflammation in the entire paw and/or anklebone. Each paw was given the scores 0–3, with the index being the sum of all four paws. The severity trait is the maximum score observed in each individual female. The onset is the number of days calculated from the first immunization to the first clinical signs of arthritis excluding unaffected animals.

### Microsatellite genotyping and linkage analysis

Tail biopsies were collected from all N2 females and the F0 generation. DNA was isolated according to a previously described protocol [12]. After screening of parental DNA with approximately 450 mouse fluorescence-labeled microsatellite markers (INTERACTIVA, Ulm, Germany), 125 informative markers were selected covering the genome. Two hundred and thirty-seven N2 mice were genotyped with markers covering all chromosomes except for the Y chromosome. PCR amplification for the markers was performed in a final volume of 10 μl in a 96-well V-bottom microtiter plate using 20 ng DNA, 10 mM KCl, 20 mM Tris–HCl, 10 mM (NH_4_)_2_SO_4_, 2 mM MgCl_2_, 0.1% Triton X-100, pH 8.8 (New England BioLabs Inc., Ipswich, MA, USA), 3 μM (10 pmol) each primer, 2 mM dNTPs (Advanced Biotechnologies, Epsom, Surrey, UK) and 0.25 U *Taq *DNA Polymerase (New England BioLabs Inc.). The following program was used to amplify the DNA: denaturation at 95°C for 3 minutes, annealing at 56°C for 45 seconds, polymerization at 72°C for 1 minute, 30 cycles of 95°C for 30 seconds, 56°C for 45 seconds and 72°C for 1 minute, and a final extension step of 7 minutes at 72°C. The PCR products were analyzed on a MegaBACE™ 1000 (Amersham Pharmacia Biotech Inc Piscataway, NJ, USA) according to the manufacturer's protocol. Data were analyzed with Genetic Profiler 1.1 (Amersham Pharmacia Biotech Inc).

Map Manager QTXb20 free software [13] was used to perform linkage analysis and the permutation test. Ninety percent of the mouse genome was within a 20 cM intermarker distance. The marker map was generated using the Kosambis map function and 1,000 permutations were performed for every phenotype (*P *< 0.05). The permutation tests were carried out to establish empirical significance thresholds for the interactions. A threshold equal to or above the 37th percentile (*P *= 0.63) was considered suggestively significant, and the level for the significant threshold was set to the 95th percentile (*P *= 0.05). Interval mapping was made with a 2 cM increase under the additive regression model in order to calculate the test statistics.

### Enzyme-linked immunosorbent assay

The adult female mice were sacrificed at 15 months of age and sera were collected. Anti-CII antibody titers in sera were analyzed by a sandwich ELISA technique [14]. In brief, CII (10 μg/ml) was coupled to immunosorbent plates overnight at 4°C. Bovine serum albumin (Sigma Chemical St Louis, MO, USA) was used for blocking, and thereafter different dilutions of control sera (purified mouse anti-CII antibodies), test sera, and positive and negative controls were added. The presence of CII-specific IgG was visualized by means of peroxidase-conjugated goat antimouse IgG.

### Statistical analysis

Statistical comparison between the different experimental groups was performed using the Mann-Whitney *U *test or Student's unpaired *t *test.

## Results

### NFR/N female mice show delayed onset and lower incidence of CIA compared with B10.Q females

**Figure 1:**
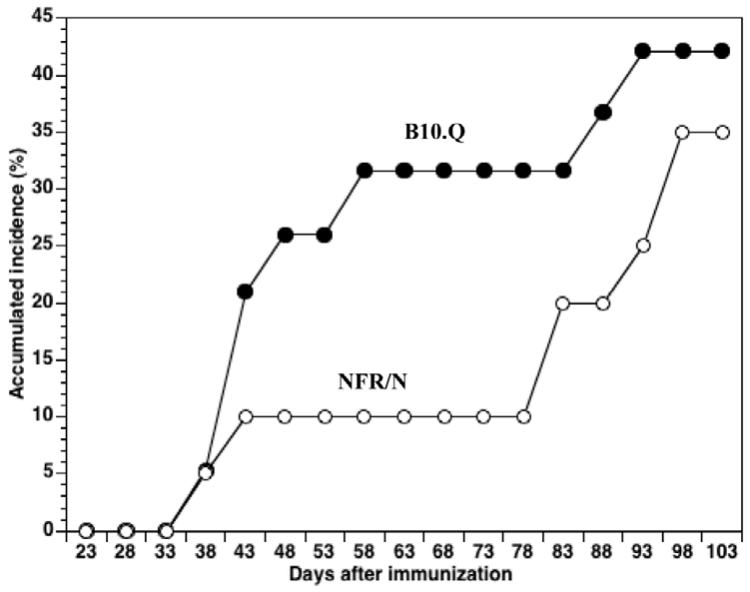
The accumulated incidence of arthritis in the parental strains (NFR/N and B10.Q) of female mice.

The onset of the disease was significantly delayed in NFR/N mice, which resulted in lower incidence in the initial phase of the disease (Table 1 and Figure 1). The appearance of the arthritis was slightly different between the two strains, NFR/N mice showing a higher frequency of swelling of the entire paw than B10.Q mice. Later in the disease course the differences in CIA frequency and severity between the strains were less obvious. However, the anti-CII titers 100 days after primary immunization (and 70 days after the booster) differed significantly between NFR/N and B10.Q female mice (see Table 1).

### Pregnancy history does not significantly affect incidence or severity of CIA in female mice of mixed genetic background

Table 2 presents the results of arthritic incidence, arthritis onset and maximum arthritic score for the female mice of the N2 generation, (NFR/N × B10.Q) × B10.Q, grouped according to the number of pregnancies they had experienced prior to immunization with CII. There was no significant difference in the incidence or severity of arthritis depending on the number of pregnancies that had been passed prior to the induction of the disease. We observed a trend towards lower severity in multiparous mice compared with mice with a history of zero to two pregnancies. The onset of diseases, however, was earlier in mice that had passed more than one pregnancy.

There was no difference in the development of the disease between female mice that have only been pregnant with MHC mismatched males (B10.RIII) and female mice that only have been pregnant with MHC matched males (B10.Q).

### New loci associated with CIA in identified old female mice

**Figure 2:**
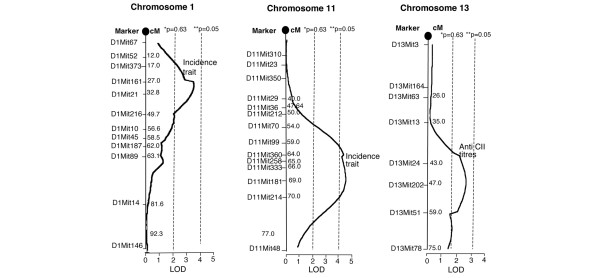
Chromosomal locations of the quantitative trait loci (QTL) for collagen-induced arthritis incidence on chromosomes 1 and 11. QTL for collagen type II (CII) antibody titers are illustrated on chromosome 13. *Suggestive level threshold (*P *= 0.63) according to the permutation test (*n *= 1,000). ** Significance level threshold (*P *= 0.05) according to the permutation test (*n *= 1,000).

The genetic linkage analysis revealed one significant quantitative trait locus (QTL) located at 64–70 cM on chromosome 11 (Table 3 and Figure 2). This locus (denoted *Cia40*) gave significant LOD scores for the traits incidence (LOD = 4.1) and 'day of onset' (LOD = 4.0), and a suggestive LOD score for the trait 'arthritis score' (LOD = 3.3). Another QTL of suggestive significance for the incidence of disease was identified around 27 cM on chromosome 1 (Figure 2). Finally, a suggestive QTL for the trait anti-CII antibody titer was identified on chromosome 13 (Figure 2).

## Discussion

Although carrying the same CIA-susceptible MHC region, the NFR/N and B10.Q mice are different in several respects. The NFR/N mouse, which is an inbred NMRI mouse of the H-2^q ^haplotype, is larger in size than the B10.Q mouse and is also known for its extraordinarily good breeding properties. The mice used in the present study, (NFR/N × B10.Q) × B10.Q females, first underwent a genetic study of reproduction, in which a number of loci associated with breeding performance were identified (Liljander M, Sällström MA, Andersson S, Wernhoff P, Andersson Å, Holmdahl R, Mattsson R; unpublished data). Since the parental strains differ in susceptibility to CIA, in spite of both having the MHC class II A beta genes [15], we wanted to test the susceptibility to inflammatory disease in mice from this cross. In fact, this situation gave an opportunity to study the genetic control of arthritis in aged multiparous females, a common situation in human RA. This susceptibility was important to investigate for various reasons. The fact that the mice differed in their reproductive history made it possible to analyze whether this would significantly affect the incidence or severity of CIA. Second, almost all the linkage analyses performed for detection of CIA-associated loci in mice have been carried out with male mice. Another reason is that the use of old multiparous mice mimicked the RA situation (older women is the major risk group). Finally, the NFR/N strain had not previously been used in linkage analyses for detection of CIA-associated loci, which opened (up) the possibility of detecting new polymorphic genes of importance in this disease.

The first conclusion from the present paper is that multiparity does not negatively influence the incidence or severity of CIA induced later in life (Table 2). This is in agreement with results from a recent study of RA in women, which similarly indicates that previous experience of pregnancy does not negatively affect the incidence or severity of RA later in life [8]. It is worth noting that the situation is quite different if pregnancy is entered during ongoing arthritis. This will normally lead to a temporary remission of the disease in both mice and humans, followed by an exacerbation phase after delivery [7,10,11].

The second conclusion from this study is that loci of possible importance for CIA have been detected, two associated with arthritis susceptibility (chromosomes 1 and 11) and one associated with anti-CII titers (chromosome 13). Some arthritis loci have previously been identified on chromosome 1: *Cia15 *at 8 cM [16], *Cia20 *at 44 cM [16] and *Cia9 *at 92 cM [17]. However, none of these loci appears to be close to the locus found on chromosome 1 in the present study. The newly identified *Cia40 *locus includes the *Ctla4 *(CD152) gene, which is a strong candidate associated with spontaneous diabetes identified in crosses between C57Bl and nonobese diabetic strains [18]. Analyses of CIA in crosses of the same backgrounds, however, did not identify a linkage to this locus, suggesting that the polymorphism underlying *Cia40 *differs from the genes associated with diabetes [18]. Interestingly, Bauer and colleagues previously identified a locus (*Cia28*) associated with anti-CII antibody production at 53 cM on chromosome 13 [19], which is approximately at the same position as where we find the linkage for this trait in the present study. It is therefore most likely that the QTL we have identified on chromosome 13 is the same as *Cia28*.

Since the possible new locus of potential interest on chromosome 1 was of suggestive significance, and since the locus identified on chromosome 13 probably is *Cia28*, we are now paying special attention to the significant QTL for CIA incidence detected at 60–70 cM on chromosome 11 (denoted *Cia40*). No other CIA genes have previously been typed in this region, but the central part of chromosome 11 is known to contain a number of inflammation loci, such as *Eae22*, *Eae6b*, *Eae23 *and *Eae7 *[20-22]. One QTL for proteoglycan-induced arthritis, which was female specific for disease onset, have also been found on chromosome 11. Although this locus (*Pgia28*) is not located in the vicinity of *Cia40*, it is worth noting [23].

The mouse chromosome 11 contains several genes that are highly conserved with human chromosome 17. Linkages for human RA have been found in this particular region [24]. Another interesting locus is *Cia5 *on the homolog rat chromosome 10, which regulates the severity of CIA and pristane-induced arthritis [25].

Interestingly, from studies with the same cross, we have previously detected a significant QTL denoted *Pregq2 *for the trait 'pregnancy frequency' in the very same region as *Cia40 *(peak at 64–70 cM on chromosome 11) (Liljander M, Sällström MA, Andersson S, Wernhoff P, Andersson Å, Holmdahl R, Mattsson R, unpublished data). This means that this region contains genes affecting the CIA incidence in multiparous mice in addition to the rate of successful deliveries in female mice. The 'pregnancy rate' in mice is defined as successful pregnancies per detected vaginal plug, a phenotype associated with early pregnancy failure, which in turn possibly could have an inflammatory cause. We cannot exclude that the same gene(s) are affecting both these traits.

## Conclusion

Our results show that multiparity does not negatively influence the incidence or severity of CIA induced later in life. Furthermore, two new loci linked to CIA susceptibility were detected on chromosome 11 (*Cia40*) and on chromosome 1. We detected on chromosome 13 a locus associated with anti-CII titers, which probably is the same as the recently reported *Cia28 *[19].

## Abbreviations

CIA = collagen-induced arthritis; CII = collagen type II; LOD = logarithm of the odds; MHC = major histocompatibility complex; PCR = polymerase chain reaction; QTL = quantitative trait locus; RA = rheumatoid arthritis.

## Competing interests

The authors declare that they have no competing interest.

## Authors' contributions

ML is responsible for genotyping, phenotyping, analyses and, together with RM, for interpretation and for writing the manuscript. M-AS and SA have contributed to collection of phenotype data. RM, ÅA, RH and ML designed the study. All authors read and approved the final manuscript.

## Figures and Tables

**Table 1:** Severity of arthritis and anti-collagen type II (anti-CII) titers in (NFR/N × B10.Q) × B10.Q female mice and parental strains

Generation	*n*	Day of onset (range)^a^	Maximum score^b^	Anti-CII titers (± standard error) (mg/ml) on day 100
F0 NFR/N	20	76.0 (11–102)	4.0 ± 1	1.53 ± 0.37^c^
F0 B10.Q	19	49.5 (12–69)	4.0 ± 1	0.87 ± 0.11
(NFR/N × B10.Q) × B10.Q	200	56.0 (8–105)	6.0 ± 0.2	1.63 ± 0.18

^a^Median value on the days of onset when calculated in all arthritic mice in the group on day 100 (maximum–minimum values in the series).

^b^Mean ± standard error of the maximum score from all arthritic mice in the respective group.

^c^Significantly higher than in B10.Q mice (*P *= 0.02 in Student's unpaired *t *test).

**Table 2:** Incidence, onset and maximum score in (NFR/N × B10.Q) × B10.Q female mice

Number of pregnancies	Number of mice	Incidence on day 50 (%)	Incidence on day 100 (%)	Day of onset (range)^a^	Maximum score^b^
0	10	10	40	62.0 (41–74)	5.5 ± 1
1	43	26	26	63.6 (31–145)	5.75 ± 0.9
2	84	13	20.5	55.3 (25–105)	6.3 ± 0.7
3	45	15	21.3	59.8 (31–76)	4.9 ± 0.8
4	18	11	27.8	51.0 (31–71)	4.2 ± 0.9

^a^Median value on the days of onset when calculated in all arthritic mice in the group (maximum–minimum values in the series).

^b^Mean (± standard error) of the maximum score from all arthritic mice in the respective group on day 100.

**Table 3:** Distribution of genotypes between affected and unaffected N2 mice at different loci linked to clinical phenotypes of collagen-induced arthritis

Trait	Locus	Location (cM)	Affected		Unaffected		LOD
				
			Homozygote for B10.Q	Heterozygote	Homozygote for B10.Q	Heterozygote	
Incidence at day 100	D1Mit161	27.0	36 (71%)	15 (29%)	70 (48%)	75 (52%)	2.8*
	D11Mit70	54.0	30 (58%)	21 (31%)	49 (34%)	96 (66%)	2.7*
	D11Mit99	59.5	35 (69%)	16 (31%)	50 (35%)	95 (65%)	4.0**
	D11Mit360	64.0	37 (73%)	14 (27%)	46 (31%)	99 (68%)	4.1**
	D11Mit258	65.0	36 (71%)	15 (29%)	49 (34%)	96 (66%)	4.1**
	D11Mit333	66.0	36 (71%)	15 (29%)	48 (33%)	97 (67%)	4.1**
	D11Mit181	69.0	37 (73%)	14 (27%)	48 (33%)	97 (67%)	4.1**
	D11Mit214	70.0	39 (76%)	12 (24%)	55 (38%)	90 (62%)	4.0*

*Suggestive significance, **significant.
